# Vimentin and PSF Act in Concert to Regulate IbeA+ *E. coli* K1 Induced Activation and Nuclear Translocation of NF-κB in Human Brain Endothelial Cells

**DOI:** 10.1371/journal.pone.0035862

**Published:** 2012-04-20

**Authors:** Feng Chi, Tao Bo, Chun-Hua Wu, Ambrose Jong, Sheng-He Huang

**Affiliations:** 1 Department of Pediatrics, Saban Research Institute of Children's Hospital Los Angeles, University of Southern California, Los Angeles, California, United States of America; 2 Division of Neonatology, The Department of Pediatrics, The Second Xiangya Hospital, Central South University, Changsha, China; Shanghai Medical College, Fudan University, China

## Abstract

**Background:**

IbeA-induced NF-κB signaling through its primary receptor vimentin as well as its co-receptor PSF is required for meningitic *E. coli* K1 penetration and leukocyte transmigration across the blood-brain barrier (BBB), which are the hallmarks of bacterial meningitis. However, it is unknown how vimentin and PSF cooperatively contribute to IbeA-induced cytoplasmic activation and nuclear translocation of NF-κB, which are required for bacteria-mediated pathogenicities.

**Methodology/Principal Findings:**

IbeA-induced *E. coli* K1 invasion, polymorphonuclear leukocyte (PMN) transmigration and IKK/NF-κB activation are blocked by Caffeic acid phenethyl ester (CAPE), an inhibitor of NF-κB. IKKα/β phosphorylation is blocked by ERK inhibitors. Co-immunoprecipitation analysis shows that vimentin forms a complex with IκB, NF-κB and tubulins in the resting cells. A dissociation of this complex and a simultaneous association of PSF with NF-κB could be induced by IbeA in a time-dependent manner. The head domain of vimentin is required for the complex formation. Two cytoskeletal components, vimentin filaments and microtubules, contribute to the regulation of NF-κB. SiRNA-mediated knockdown studies demonstrate that IKKα/β phosphorylation is completely abolished in HBMECs lacking vimentin and PSF. Phosphorylation of ERK and nuclear translocation of NF-κB are entirely dependent on PSF. These findings suggest that vimentin and PSF cooperatively contribute to IbeA-induced cytoplasmic activation and nuclear translocation of NF-κB activation. PSF is essential for translocation of NF-κB and ERK to the nucleus.

**Conclusion/Significance:**

These findings reveal previously unappreciated facets of the IbeA-binding proteins. Cooperative contributions of vimentin and PSF to IbeA-induced cytoplasmic activation and nuclear translocation of NF-κB may represent a new paradigm in pathogen-induced signal transduction and lead to the development of novel strategies for the prevention and treatment of bacterial meningitis.

## Introduction

Over the past few years, studies of the common neonatal bacterial meningitis caused by *Escherichia coli* K1 revealed the importance and significance of IbeA, a major virulence determinant during the early stage of neonatal *E. coli* infection [1], and its interactions with host factors in brain microvascular endothelial cells (BMEC), including vimentin (primary receptor), polypyrimidine tract-binding protein (PTB)-associated splicing factor (PSF) (co-receptor), and related signaling molecules (*e.g.*, caveolin-1, ERK, and CaMKII) [2]–[6]. The *ibeA* gene locus (GimA) is unique to *E. coli* pathogens but is not present in nonpathogenic *E. coli* K12 strains [7]–[8]. It has been widely used as a genetic marker in the genotyping of *E. coli* strains isolated from various host and environmental sources [1], [9]–[14]. The *ibeA* locus is able to modulate expression of several virulence factors (*e.g.*, *aatA*, *fim*, *ibeB*, *ompA* and biofilm-associated genes) and predominantly contributes to *E. coli* K1-caused early-onset human neonatal sepsis and meningitis by inducing both pathogen penetration and polymorphonuclear leukocyte (PMN) transmigration across the blood-brain barrier (BBB), which consists mainly of BMEC [1], [15]–[17]. IbeA is positively associated with multidrug resistance [14], [18]. The specific IbeA–BMEC surface protein interaction and subsequently induced signal transduction were shown to be essential for *E. coli* K1 invasion [19]–[20]. Two IbeA-binding proteins have been identified: vimentin, which is constitutively present in the surface of human BMECs (HBMECs), and PSF, which is inducibly expressed in both mesenchymal (endothelium) and non-mesenchymal (epithelium) cells [2]–[3]. IbeA-induced signaling through its binding proteins vimentin as well as PSF is required for meningitic *E. coli* K1 penetration across the blood-brain barrier (BBB), which is one of the hallmarks of bacterial meningitis [4]–[6]. Vimentin is a well-known marker for mesenchymal cells such as endothelial cells [2]. Epithelial–mesenchymal transition (EMT) processes are usually associated with embryonic development and the malignant conversion of epithelial tumour cells [21]. This protein also contributes to the adhesive or invasive phenotype of microbial pathogens, including *P. multocida* and African swine fever virus [2]. Our previous studies have shown that CaMKII-induced phosphorylation of the vimentin head domain and the vimentin-binding domain of ERK are necessary for IbeA+ *E. coli* K1-mediated invasion of BMEC. PSF is mainly present in the nucleus, but it can be translocated to the cytoplasm and cell surface [2]. It has multiple functions, including binding of nucleic acids (DNA and RNA) and proteins, DNA pairing, promotion of pre-mRNA splicing and transcriptional regulation. Besides these properties, PSF may contribute to regulation of protein kinase C [22] and ERK [23]. Our recent study demonstrated that PSF could contribute to IbeA-mediated *E. coli* K1 invasion of HBMECs in a manner dependent on lipid rafts [2], [4]. Upon stimulation with *E. coli* K1 or IbeA-coated beads, vimentin and PSF are recruited to the raft microdomains, suggesting that that lipid rafts in HBMECs serve as a common platform for both proteins contributing to IbeA-induced virulence. However, it is unknown how vimentin and PSF cooperatively contribute to IbeA-induced cellular signaling and bacterial invasion.

Our recent studies have demonstrated that the IbeA/Vim-mediated signaling is essential for NF-κB activation and PMN transmigration across the BBB, two additional hallmark features of bacterial meningitis [5], [24]–[26]. There is a dual role for PMN recruitment to the CNS. PMN transmigration across the BBB is not only a key aspect of the protective response against invading pathogens, but leukocytes also contribute importantly to the deleterious effects of inflammation on the CNS tissues in bacterial meningitis, which results in devastating neurologic sequelae [27]. Both *in vitro* and *in vivo* studies suggest that IbeA and vimentin are essential for *E. coli* K1-induced PMN transmigration across the BBB. Vimentin on both endothelial cells and PMN is able to form an anchoring structure at the site of contact between these two cell types. Vimentin contributes to PMN transmigration across the BBB in response to meningitic infection in a manner substantially similar to the traversal of lymphocytes across peripheral endothelial cells [28]. *E. coli*-induced PMN transmigration could be markedly inhibited by withaferin A, a dual inhibitor of vimentin and proteasome. IbeA-induced PMN migration could be also blocked by PS-341 (bortezomib), a proteasomal inhibitor, and correlated with up-regulation of vimentin, ICAM-1, and CD44 expression through proteasomal regulation of NF-κB activity [5]. Vimentin could stabilize adhesion molecules and Scribble by protecting them from proteasomal degradation [5]. These findings suggest that IbeA is able to induce nuclear translocation of NF-κB and upregulate vimentin that are correlated with *E. coli* K1-stimulated PMN transendothelial migration and inflammatory response. Currently, it remains elusive how IbeA contributes to both bacterial invasion and PMN transmigration through its interactions with vimentin/PSF and upregulation of NF-κB activity.

The NF-κB signal transduction pathway, which is the master regulator of the innate immunity, plays important roles in maintaining cell homeostasis/differentiation, and regulating the host response to microbial infections [29]. NF-κB proteins consist of five different members, p65/RelA, c-Rel, RelB, NF-κB2/p52, and NF-κB1/p50, sharing a Rel homology domain that mediates DNA binding and dimerization [30]. In resting cells, NF-κB is trapped in the cytoplasm by inhibitory IκB proteins. The NF-κB activation process is induced by phosphorylation of serine residues on the IκB proteins, which are then subjected to ubiquitination and proteasomal degradation. Deregulated activity of this pathway has been linked to the progression of a number of human diseases, including cancers and microbial infections [29]. For example, NF-κB activation has been shown to contribute to *N. meningitidis* invasion of epithelial cells [31] and *H. pylori*-induced inflammation in gastric epithelium [32]. NF-κB in the CNS is activated during bacterial meningitis [24]. NF-κB inhibitors have been found to reduce the CNS inflammation and to protect rat brains from inflammatory injury following transient focal cerebral ischemia [33] and pneumacoccal meningitis [25]. Our studies suggest that IbeA induces all three of the hallmark features of bacterial meningitis: NF-κB activation, pathogen invasion and PMN transmigration across the BBB [4]–[6]. The underlying mechanisms for these pathogenicities, however, require further investigation. Recently, vimentin has emerged as a signaling platform of a number of critical proteins at the cell surface, which contribute to the invasive phenotypes of cancer, *E. coli*-induced pathogenicities, and cell signaling [5]–[6], [34]. PSF's main function as a nuclear protein is to contribute to the promotion of pre-mRNA splicing and transcriptional regulation [3], [35]. These findings suggest that vimentin and PSF may cooperatively contribute to the early and late signaling events induced by IbeA, allowing us to speculate that NF-κB activation is mainly mediated by vimentin and that PSF plays a major role in both cytoplasmic activation and nuclear translocation of NF-κB. To clarify these issues, we investigated in this study the role of vimentin and PSF in IbeA-induced NF-κB signaling.

## Results

### IbeA-induced *E. coli* K1 invasion, PMN transmigration and IKK/NF-κB activation are blocked *in vitro* and *in vivo* by Caffeic acid phenethyl ester (CAPE), an inhibitor of NF-κB

**Figure 1:**
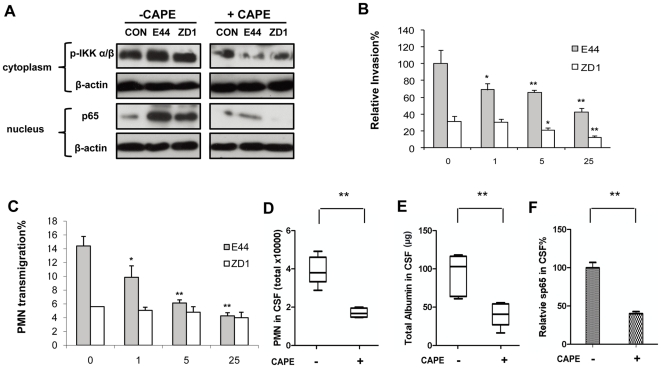
Effects of CAPE on IbeA+ *E. coli* K1-induced NF-κB activation and pathogenicities *in vitro* and *in vivo*. (**A**) IbeA+ *E. coli* K1 induced NF-κB activation in HBMECs was suppressed by CAPE. HBMECs were incubated with or without the NF-κB inhibitor CAPE (25 µM) for 30 min before stimulation with E44 or ZD1 (10^7^/mL). IKK α/β phosphorylation (p-IKK α/β) in cytoplasmic fractions and NF-κB (p65) in nuclear fractions was examined after 2 h of stimulation with *E. coli* strains. The β-actin in both fractions was detected as internal loading controls. CON, control without *E. coli* stimulation. (**B–C**) Effects of CAPE (0–25 µM) on IbeA+ *E. coli* K1 penetration and PMN transmigration across HBMECs were examined. HBMECs were incubated with various concentrations of CAPE for 1 h before the invasion and PMN transmigration assays. (**B**). *E. coli* (10^7^ CFU) were added to the HBMEC monolayers after CAPE treatment. Invasion assays were carried out as described in the Materials and Methods. (**C**) The CAPE-pretreated HBMECs were stimulated with *E. coli* (10^6^ CFU) in the lower chamber for 2 h and incubated with PMN (10^6^) in the upper chamber at 37°C for another 4 h. All assays were performed in triplicates. Results for invasion are expressed as relative invasion compared to the positive control without drug treatment (100%). Results for PMNT are expressed as the percentage of leukocyte transmigration of the total added. Both the invasion and PMNT assays were done with E44 (black column) and ZD1 (white column). *E. coli* meningitis was induced in neonatal mice with or without CAPE treatment (n = 5) as described in Methods and Materials. (**D**) Recruitment of PMN into the CSF; (**E**) Flux of albumin into the CNS; and (**F**) Levels of soluble NF-κB (p65) in CSF. The significant differences with regard to the controls without CAPE treatment were marked by asterisks (*P<0.05; **P<0.01).

Our previous studies suggest that NF-κB activation may play a key role in IbeA-induced bacterial invasion and PMN transmigration [4]–[5]. However, the underlying mechanism is unclear. In order to dissect this issue, CAPE, a specific inhibitor of NF-κB, was used to determine the role of NF-κB signaling in both IbeA+ *E. coli* K1 induced Invasion and PMN transmigration. As shown in Fig. 1A, IbeA+ *E. coli* (E44) could significantly increase phosphorylation of IKK α/β and NF-κB translocation to the nucleus when compared to the IbeA deletion mutant ZD1 and the control without treatment. These cellular effects were remarkably reduced by inhibition of NF-κB signaling with CAPE. CAPE could also significantly block IbeA+ *E. coli* K1 (E44)-induced invasion of HBMECs and PMN transmigration in a dose-dependent manner (Figure 1B and 1C). However, the dose-dependent effects on PMN transmigration could not be observed with the *ibeA* mutant ZD1 under the same experimental conditions (Figure 1C). Residual effects of CAPE-mediated inhibition on ZD1 were also observed, indicating that other virulence factors might be regulated by NF-κB. To further confirm these *in vitro* findings, mice were pre-treated with CAPE for 3 days and then infected with E44. As shown in Fig. 1D–F, CAPE was able to significantly reduce the levels of PMN, albumin and NF-κB in CSF. Overall, these findings suggest that the NF-κB signaling pathway plays an important role in IbeA+ *E. coli* K1 induced Invasion and PMN transmigration across HBMECs.

### MEK/ERK signaling mediates IKK complex and NF-κB activation in response to IbeA+ *E. coli* K1 infection of HBMECs

**Figure 2:**
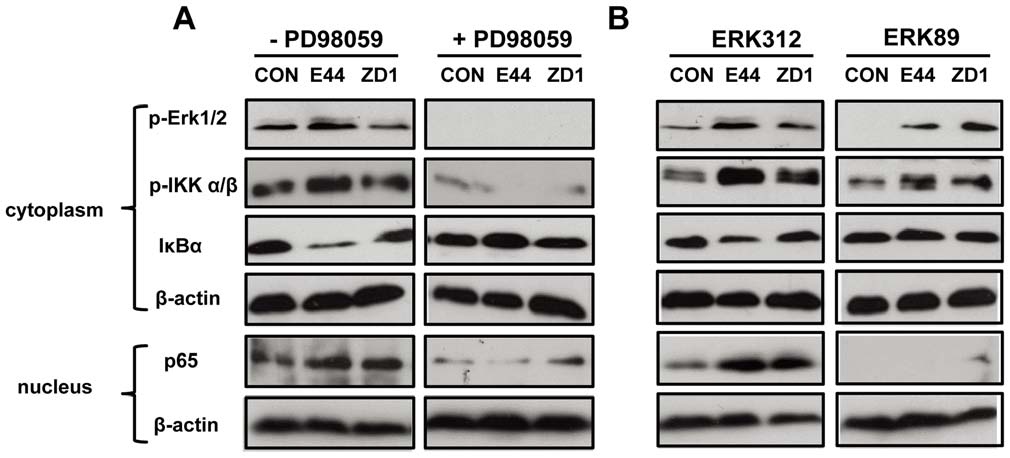
Inhibition of IbeA+ *E. coli*-induced IKK phosphorylation and NF-κB activation by MEK/ERK inhibitors. (**A**) HBMECs were incubated with or without PD098059 (50 µM) for 60 min before stimulation with E44 or ZD1 (10^7^/ml). (**B**) HBMECs were incubated with or without ERK89 (vimentin-binding domain, 25 µg/ml) and ERK312 (control peptide, 25 µg/ml) for 60 min before infection with E44 or ZD1 (10^7^/ml). In both (**A**) and (**B**), ERK1/2 phosphorylation (p-Erk1/2), IKK α/β phosphorylation (p-IKK α/β) and IκBα degradation were examined in cytoplasmic fractions after 30 min of stimulation with *E. coli* K1 strains. NF-κB (p65) translocation to the nucleus was examined in nuclear fractions after 2 h of infection with *E. coli* K1 strains. β-actin in both fractions was detected as internal loading controls. CON, control without bacterial stimulation.

The MAPK/ERK signaling pathway, a chain of proteins communicating a signal from a receptor on the cell surface to the DNA in the nucleus, has been implicated in NF-κB activation in response to Ras activation [36] or anthracycline drug treatment [37]. We have demonstrated that ERK phosphorylation is required for IbeA-induced *E. coli* K1 invasion [4]. Binding of vimentin to phospho-ERK can protect the activated kinase from dephosphorylation [38]. PSF contributes to IGF-I-mediated regulation of ERK through association and dissociation of p-ERK [23]. Thus, we sought to determine whether IbeA-ERK activation could also promote activation of NF-κB through the MEK/ERK signaling pathway. PD098059, an ERK inhibitor, was first used to examine the role of ERK phosphorylation in IbeA+ *E. coli*-induced NF-κB activation. Figure 2A showed that E44 could increase phosphorylation of Erk1/2 and IKK α/β, degradation of IκBα after 30 min of incubation, and NF-κB translocation to the nucleus after 2 h of infection when compared to the IbeA deletion mutant ZD1 and the control without treatment. We then used synthetic ERK peptides ERK89 (vimentin-binding domain) and ERK312 (non-binding peptide) to examine whether the vimentin-binding domain of ERK was essential for IbeA+ *E. coli*-induced NF-κB activation. As shown in Figure 2B, ERK89 was able to block the Erk1/2 and IKK α/β phosphorylation, IκBα degradation, and NF-κB translocation to the nucleus when compared to the control peptide ERK312. It suggests that the regulation of ERK by vimentin is important for IbeA+ *E. coli*-induced NF-κB activation based on its protection effect on ERK phosphorylation. Taken in consideration together, these results demonstrated that the MEK/ERK signaling pathway is critical for IbeA+ *E. coli* K1-induced cytoplasmic activation and nuclear translocation of NF-κB.

### IbeA+ *E. coli* K1 induced NF-κB activation is vimentin-dependent

**Figure 3:**
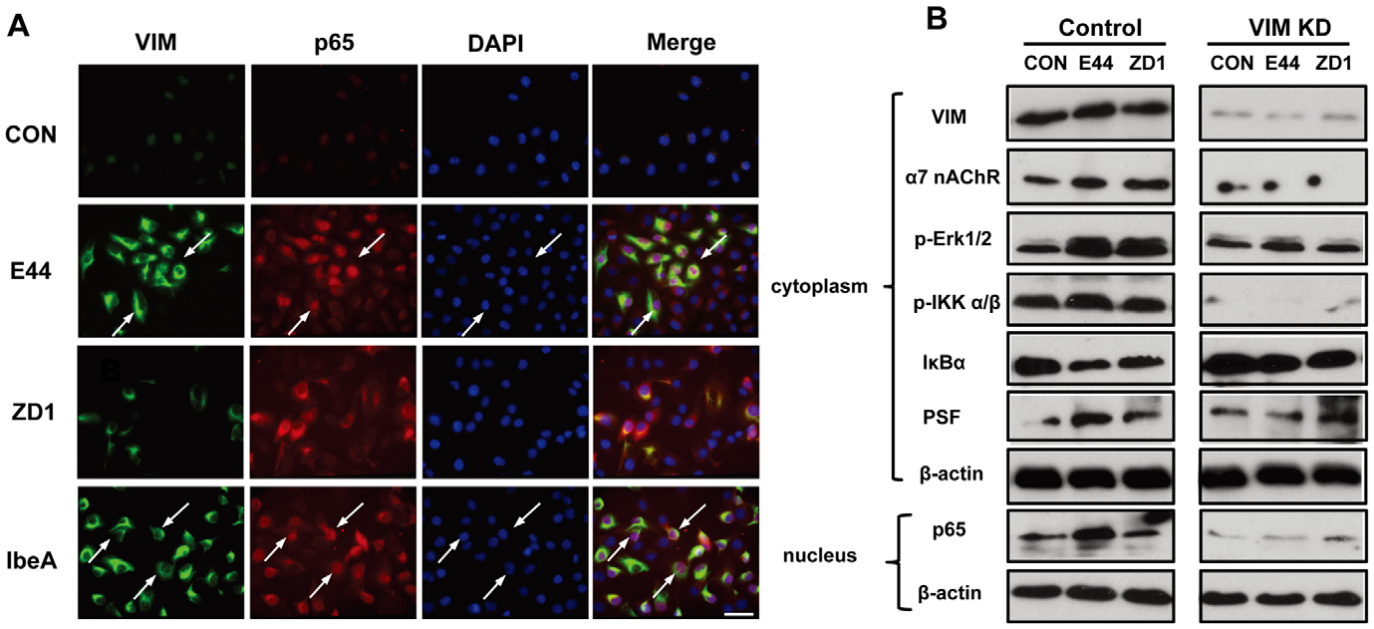
Role of vimentin in IbeA+ *E. coli* K1-induced NF-κB activation. (**A**) Immunofluorescence microscopy was used to examine the correlation between vimentin reorganization and NF-κB translocation to the nucleus after 2 h of stimulation with IbeA protein (0.1 µg/ml), E44 or ZD1 (25 MOI). HBMECs were triple-stained with the V9 antibody against vimentin conjugated to FITC (green), the rabbit antibody against NF-κB (p65) conjugated to rhodamine (red), and DAPI (blue). The merged images are shown in the right-hand panels (Merge). Arrows indicated cells with colocalization of vimentin and NF-κB (p65) Scale bar, 50 µm. (**B**) Blockage of IbeA+ *E. coli* K1-induced NF-κB activation in HBMECs by siRNA-mediated knockdown of vimentin. HBMECs were transfected with vimentin or control siRNA as described in Materials and Methods. After 24 h incubation, the cells were treated with E44 or ZD1 (10^7^/ml) for 30 min or 2 h. Vimentin (VIM), α7 nAChR, ERK1/2 phosphorylation (p-Erk1/2), IKK α/β phosphorylation (p-IKK α/β), IκBα degradation, and PSF re-localization were examined in cytoplasmic fractions after 30 min of stimulation with *E. coli* K1 strains. NF-κB (p65) translocation to the nucleus was examined in nuclear fractions after 2 h of incubation with *E. coli* K1 strains. β-actin in both fractions was detected as internal loading controls. Control: HBMECs transfected with control siRNA; VIM KD: HBMECs transfected with vimentin siRNA; UNT: Untreated HBMECs.

Since IbeA+ *E. coli* K1 was able to induce extensive vimentin reorganization in HMBECs [4], we assumed that this change was involved with IbeA+ *E. coli* K1-induced NF-κB activation. In order to dissect this issue, immunofluorescence microscopy was used to examine the colocalization of vimentin and NF-κB. Figure 3A showed that there were very few changes in vimentin reorganization and NF-κB translocation occurring in the control without any treatment or HBMECs treated with the IbeA deletion mutant ZD1 after 2 hours of incubation, while E44 and IbeA could strongly induce both vimentin rearrangements and NF-κB translocation to the nucleus. The cytoplasmic NF-κB is clustered and colocalized with vimentin rearrangements (indicated by arrows). We used siRNA-mediated knockdown to further examine the role of vimentin in IbeA+ *E. coli* K1-induced NF-κB activation. As shown in Figure 3B, cytoplasmic levels of vimentin, α7 nAChR and PSF were significantly reduced in vimentin siRNA-transfected cells. Erk1/2 and IKK α/β phosphorylation, IκBα degradation, and NF-κB translocation to the nucleus were significantly reduced in all treatments of HBMECs transfected with vimentin siRNA. These results suggest that vimentin is required for IbeA+ *E. coli* K1-induced NF-κB activation.

### Time course analysis of IbeA-induced cytoplasmic activation and nuclear translocation of NF-κB

**Figure 4:**
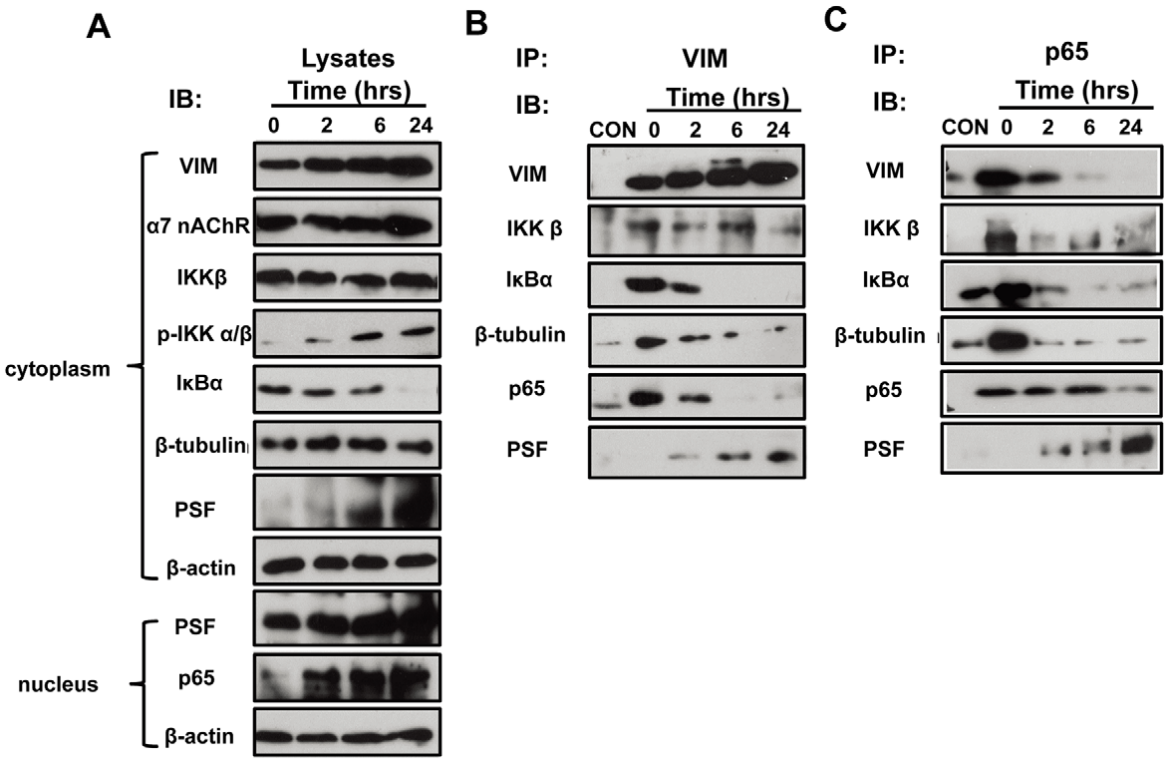
Time course analysis of IbeA-induced cytoplasmic activation and nuclear translocation of NF-κB. HBMECs ware incubated with the IbeA protein (0.1 µg/ml) for 2, 6, and 24 h, respectively, and then the cytoplasmic and nuclear fractions were extracted. The cytoplasmic fractions were immunoprecipitated (IP) with the V9 anti-vimentin antibody and the rabbit anti-NF-κB (P65) antibody as described in Materials and Methods. The cytoplasmic and nuclear lysates (**A**), vimentin Co-IP complexes (**B**), and NF-κB (p65) Co-IP complexes (**C**) were subjected to western blot using the antibodies as described in Materials and Methods. CON: the IP control without primary antibodies incubation; 0 h: the control HBMECs without IbeA stimulation.

As shown in our previous studies [4]–[5], vimentin and PSF may play sequential roles in the early and late signaling events induced by IbeA. Because the mechanisms by which IbeA induces signaling are still poorly understood and the molecular players responsible for the initiation of NF-κB activation and subsequent nuclear translocation are not clear, we performed time course analysis of IbeA-induced NF-κB activation and translocation. HBMECs were treated with IbeA at different time points (2–24 h). Since vimentin is the primary receptor of IbeA, we first examined its interactions with PSF, NF-κB and other signaling molecules by co-immunoprecipitation (Co-IP) of vimentin and NF-κB (p65). The protein lysates from cytoplasmic fractions were examined and used as positive controls for Co-IP assays. As shown in Figure 4A, IbeA could increase expression of vimentin, α7 nAChR, and PSF, phosphorylation of IKK α/β, and degradation of IκBα in the cytoplasm. Meanwhile, IbeA also significantly enhance the protein content of p65 in nuclear fractions and PSF in both cytoplasmic and nuclear fractions in a time-dependent manner. The Co-IP assay with a mouse monoclonal antibody against vimentin showed that proteins in the Co-IP complex including NF-κB (p65), IκB, and β-tubulin decreased in a time-dependent manner (Figure 4B), while IbeA could markedly enhance vimentin expression. However, PSF was dramatically increased upon prolonged stimulation with IbeA. These results suggested that there was an increased dissociation of vimentin with IκBα, NF-κB and β-tubulin, meanwhile an association of vimentin with PSF was increased throughout the time course of stimulation with IbeA. Similar patterns of changes in vimentin, IκBα, NF-κB, β-tubulin and PSF were observed in the Co-IP assay with the rabbit anti-p65 antibody (Figure 4C). These findings suggest that vimentin and PSF cooperatively contribute to the early (cytoplasmic activation) and late (nuclear translocation) events of IbeA-induced NF-κB signaling. It appears that vimentin forms a complex with IκB, NF-κB and tubulins in the resting cells. A dissociation of this complex and a simultaneous association of PSF with NF-κB could be induced upon the prolonged stimulation with IbeA.

### Vimentin head domain is required for IbeA-induced NF-κB activation and interaction with β-tubulin

**Figure 5:**
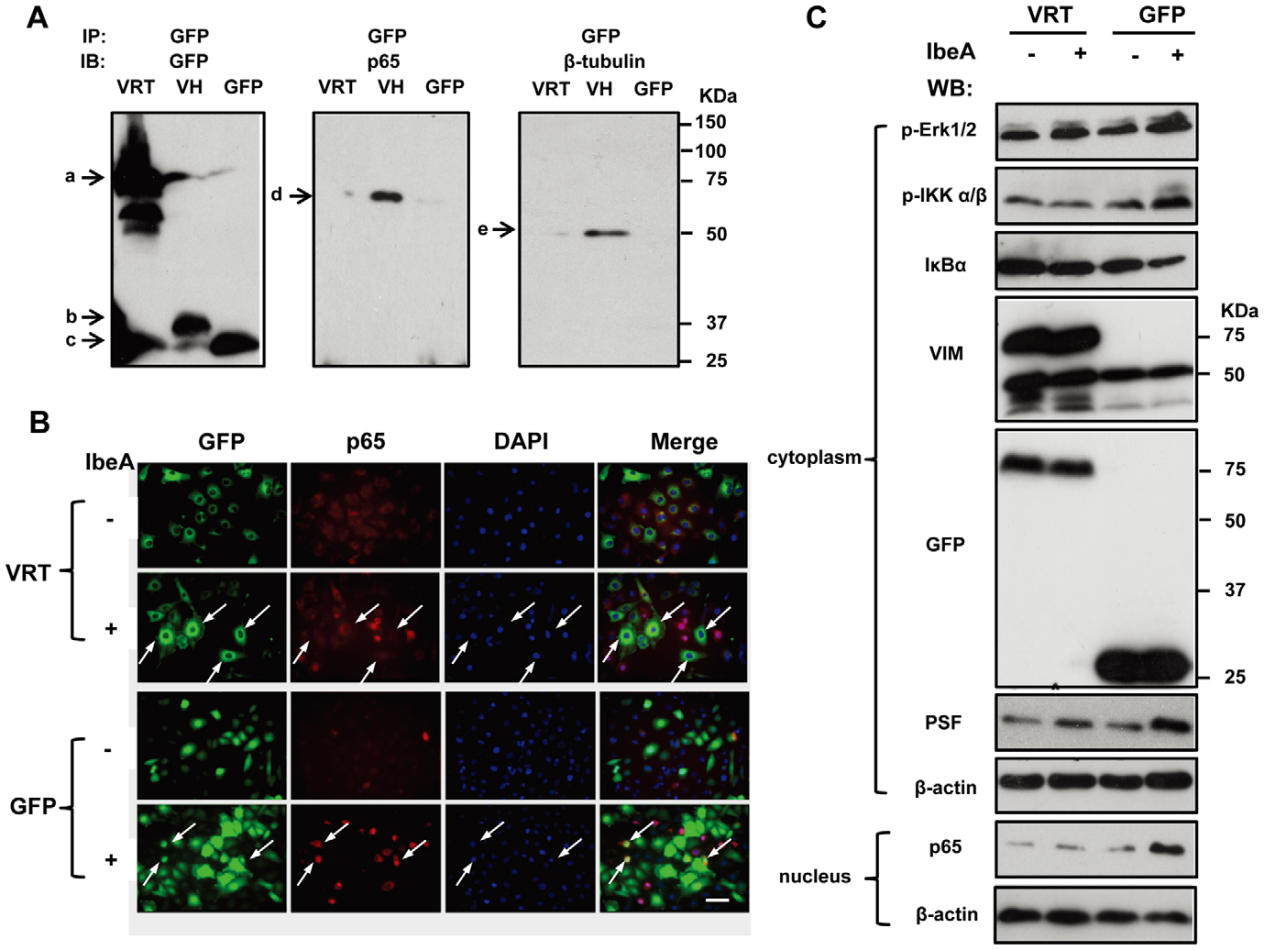
Effects of vimentin head domain deletion on IbeA-induced NF-κB activation and interaction with β-tubulin. (**A**) The cytoplasmic fractions of the GFP–VRT, GFP-VH and GFP transductants were extracted and immunoprecipitated (IP) using the mouse anti-GFP antibody as described in Materials and Methods. The GFP-IP complexes were subjected to Western blotting using the rabbit polyclonal antibodies against GFP, NF-κB (P65), and β-tubulin. Band a, GFP–VRT (72 kDa); band b, GFP-VH (37 kDa); band c, GFP (27 kDa); band d, NF-κB (P65), (65 kDa); and band e, β-tubulin, (50 kDa). (**B**) Immunofluorescence images of the GFP–VRT and GFP transductants incubated with or without the IbeA protein (0.1 µg/ml) for 2 h. The cells were double-stained with the rabbit antibody against NF-κB (p65) conjugated to rhodamine (red), and DAPI (blue). Arrows indicate cells with NF-κB (P65) translocation to the nucleus, which was increased in the GFP transductants and reduced in GFP-VRT-transduced HBMECs upon stimulation with IbeA. Scale bar, 50 µm. (**C**) Western blot of the transduced HBMECs treated with the IbeA protein (0.1 µg/ml). ERK1/2 phosphorylation (p-Erk1/2), IKK α/β phosphorylation (p-IKK α/β), IκBα degradation, vimentin (VIM), GFP and PSF re-localization were examined in cytoplasmic fractions after 30 min of IbeA stimulation. NF-κB (p65) translocation to the nucleus was examined in nuclear fractions after 2 h of IbeA incubation. β-actin in both fractions was detected as internal loading controls.

To dissect the interaction between vimentin and NF-κB (p65) or β-tubulin, we generated two lentivirus constructs with GFP fusion, which express the vimentin head domain (GFP-VH) in N-terminal and the vimentin rod and tail domains with a VH deletion (GFP-VRT) in C-terminal as described in Materials and Methods [4]. The lysates of the lentivirus transduced HBMECs were first subjected to Western blot to examine expression of the vimentin fragment constructs. As shown in Figure S1A, a 72-kDa protein (band a) in the GFP–VRT transductant, a 37-kDa protein (band b) in the GFP–VH transductant, and a 27-kDa protein (band c) in the GFP transductant were detected by a rabbit antibody against GFP. Because the two anti-vimentin antibodies recognize the C-terminal region (V9) and the N-terminal region (H84) [39], respectively, we used them to detect the endogenous expression of vimentin fragments. The results showed a 72-kDa protein (band a) in the GFP–VRT transductant besides the native vimentin of 55-kDa (band d) in all transduced cells with the V9 antibody, and a 37-kDa protein (band b) in the GFP–VH transductant besides the native vimentin of 55-kDa (band d) in all transduced cells with the H84 antibody. Since the Co-IP assays indicated that vimentin could form a complex with IκB, NF-κB (p65) and β-tubulin in the resting cells, we then performed a Co-IP assay with a mouse monoclonal antibody against GFP tag using all the transduced HBMECs without any treatment. The GFP-IP complexes were detected with a rabbit antibody against GFP, a rabbit antibody against NF-κB (p65), and a rabbit antibody against β-tubulin (Figure 5A). The results showed a similar pattern to those in Figure S1A using the GFP antibody, except some cleavages of GFP-VRT and GFP-VH, perhaps due to the instability of the GFP fusion proteins. Interestingly, both NF-κB (p65) and β-tubulin bind to the N-terminal region, suggesting that the vimentin head domain contributes to IbeA-induced NF-κB signaling in a coordinated manner with microtubules. In order to further examine the role of the VH domain in IbeA-induced NF-κB activation, the immunofluorescence microscopy was next used to examine the effects of the VH deletion on NF-κB (p65) translocation to the nucleus. The GFP transductant was taken as a positive control. As shown in Figure 5B, IbeA-induced NF-κB (p65) translocation to the nucleus occurred in both the non-transduced HBMECs and GFP-transduced HBMECs (indicated by arrows), suggesting that the GFP lentivirus transduction did not affect the NF-κB pathway. However, the GFP-VRT transductant was deficient in NF-κB (p65) translocation to the nucleus (indicated by arrows), indicating that the vimentin head domain is essential for IbeA-induced NF-κB activation. The same results were also obtained with these transductants upon IbeA+ *E. coli* (E44) infection, but these events could not occur in the untreated controls of the GFP-VRT and GFP transductants or the cells treated with ZD1. This further confirmed the key role of the VH domain in this process (Figure S1C). Western blot showed that the VH domain deletion significantly inhibited the NF-κB pathway, including Erk1/2 and IKK α/β phosphorylation, IκBα degradation, and NF-κB translocation to the nucleus upon stimulation with both the IbeA protein (Figure 5C) and IbeA+ *E. coli* (E44), but it did not occur in the cells treated with ZD1 (Figure S1B). To further investigate the interaction between vimentin and NF-κB-IκB complex, a pull-down assay was performed using purified His-vimentin and Ni-NTA resin column after incubation with total cell lysates of HBMEC. As Shown in Figure S2, IκBα (Figure S2D) could directly bind to vimentin when compared to the control with the Ni-NTA resin only. All these findings suggest the involvement of vimentin in IκBα binding and the role of the vimentin head domain in IbeA-induced NF-κB activation.

### Microtubule network is required for IbeA+ *E. coli* K1-induced NF-κB activation

**Figure 6:**
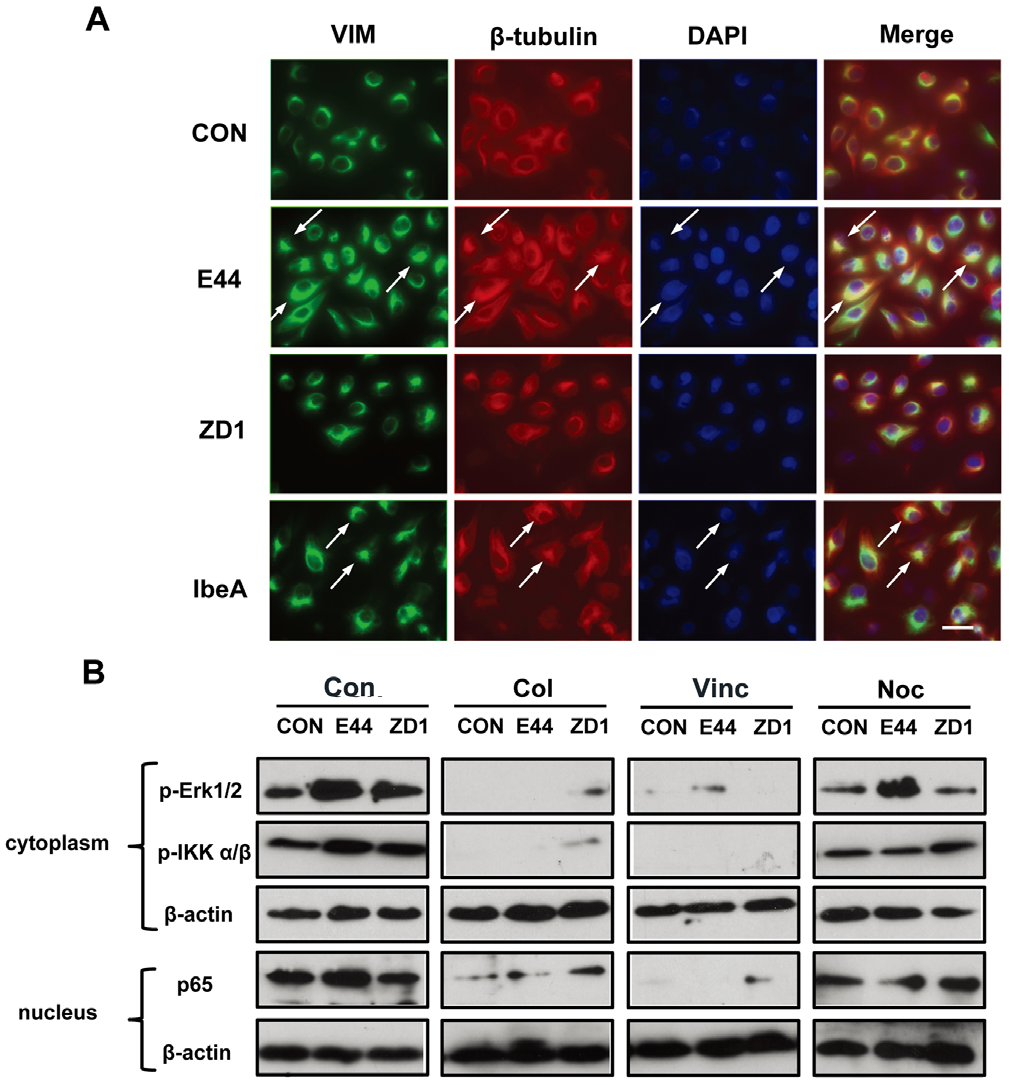
β-tublulin is required for IbeA+ *E. coli* K1-induced NF-κB activation. (**A**) IbeA− and IbeA+ *E. coli* K1-induced β-tubulin/vimentin clustering and colocalization. Immunofluorescence microscopy was used to examine the clustering and reorganization of vimentin and β-tubulin after 2 h of incubation with the IbeA protein (0.1 µg/ml), E44 or ZD1 (25 MOI). HBMECs were triple-stained with the V9 antibody against vimentin conjugated to FITC (green), the rabbit antibody against β-tubulin conjugated to rhodamine (red), and DAPI (blue). The merged images are shown in the right-hand panels (Merge). Arrows indicated cells with colocalization between vimentin and β-tubulin around the perinuclear region. Scale bar, 50 µm. (**B**) Blockage of IbeA+ *E. coli* K1-induced cytoplasmic activation and nuclear translocation of NF-κB (p65) in HBMECs by the microtubule inhibitors. HBMECs were incubated with or without colchicines (Col, 2 µM), vincristine (Vin, 1 µM), nocodazole (Noc, 25 µg/ml) for 60 min before stimulation with E44 or ZD1 (10^7^/ml). Phosphorylation of ERK1/2 (p-Erk1/2) and IKK α/β (p-IKK α/β) was examined in cytoplasmic fractions after 30 min of *E. coli* K1 treatment. NF-κB (p65) translocation to nucleus in nuclear fractions was examined after 2 h of *E. coli* K1 incubation. β-actin in both fractions was detected as internal loading controls. CON, control without bacterial stimulation.

The GFP-tag Co-IP assay demonstrated that β-tublulin could bind to the vimentin head domain, suggesting that these two kinds of cytoskeletal filaments may coordinately contribute to NF-κB signaling. Microtubules have been implicated in NF-κB activation and translocation [40]–[42]. Thus, we further examined the role of the microtubule network in IbeA-induced NF-κB activation. Immunofluorescence microscopy was used to examine colocalization of vimentin and β-tubulin upon stimulation with E44, ZD1 and IbeA. As shown in Figure 6A, both E44 and IbeA could strongly induced β-tubulin reorganization, which was partially colocalized with vimentin clusters around the perinuclear region (arrows indicated), while very few colocalization areas were detected in unstimulated cells or cells treated with ZD1. To confirm whether the microtubule network participates in IbeA-induced NF-κB signaling, microtubule inhibitors, including colchicine, vincristine, and nocodazole, were used to examine their inhibitory effects on IbeA-induced NF-κB activation. As shown in Figure 6B, all these microtubule inhibitors could block Erk1/2 and IKK α/β phosphorylation, and NF-κB translocation to the nucleus, indicating that the microtubule network plays an important role IbeA+ *E. coli*-induced cytoplasmic activation and nuclear translocation of NF-κB.

### IbeA+ *E. coli* K1-induced pathogenicities, phosphorylation of ERK/IKK and nuclear translocation of NF-κB were significantly blocked by siRNA-mediated knockdown of PSF

**Figure 7:**
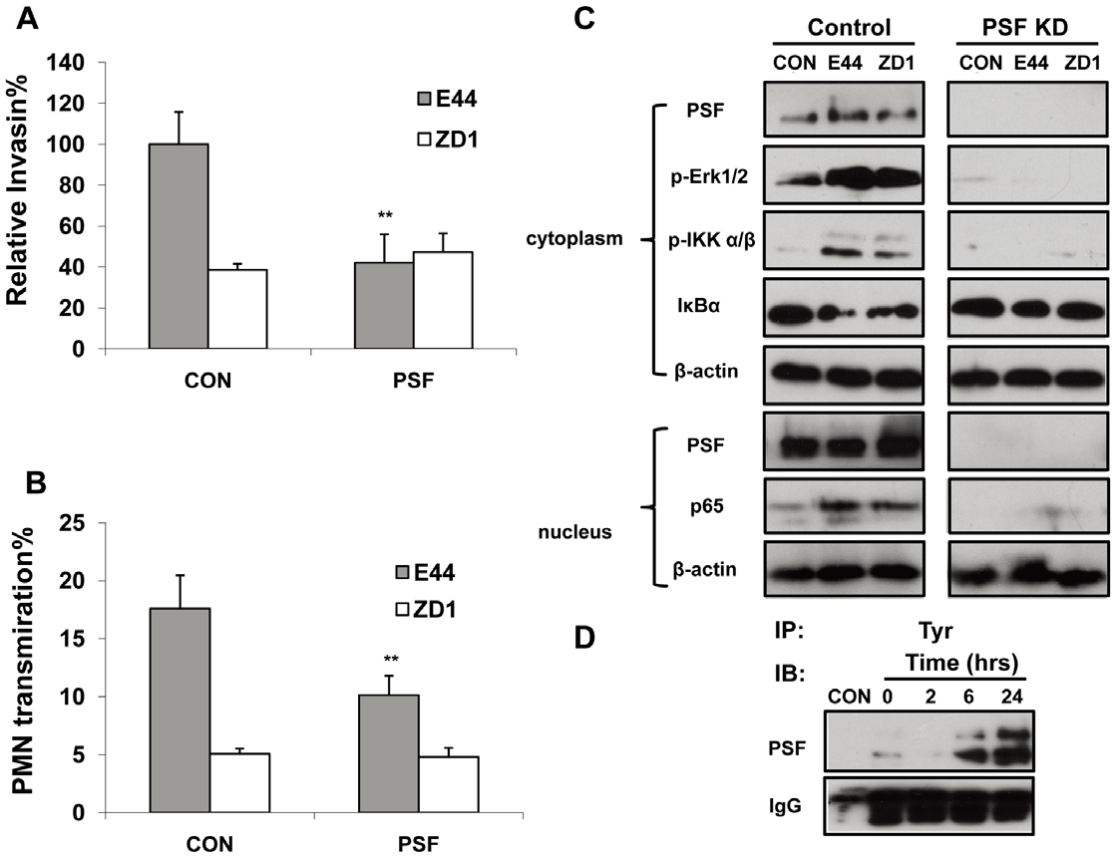
Inhibition of IbeA+ *E. coli* K1-induced pathogenicities, phosphorylation of ERK/IKK and nuclear translocation of NF-κB by knockdown of PSF. HBMECs were transfected with PSF or control siRNA as described in Materials and Methods. IbeA+ *E. coli* K1 penetration (**A**) and PMN transmigration (**B**) across siRNA-transfected HBMECs were performed as described in the Materials and Methods. Both invasion and PMN transmigration assays were performed in triplicates. Results for invasion are expressed as a relative percentage compared to the penetration rate of E44 in the siRNA control (CON) (100%). Results for PMN transmigration are expressed as the percentage of PMN transmigration of total PMNs. The control siRNA-transfected HBMECs infected with E44 and ZD1 were used as the controls (panels A and B). The significant differences regarding to the control were marked by asterisks (*P<0.05; **P<0.01). (**C**) After transfection, the cells were stimulated with E44 or ZD1 (10^7^/ml) for 30 min or 2 h. PSF re-localization, p-Erk1/2, p-IKK α/β and IκBα degradation were examined in cytoplasmic fractions after 30 min of stimulation with *E. coli* K1 strains. NF-κB (p65) and PSF in nuclear fractions were examined after 2 h of incubation with *E. coli* K1 strains. β-actin in both fractions was detected as internal loading controls. Control: HBMECs transfected with control siRNA; PSF KD, HBMECs transfected with PSF siRNA; UNT: Untreated HBMECs. (**D**) Time course analysis of IbeA-induced tyrosine phosphorylation of PSF. HBMECs were incubated with the IbeA protein (0.1 µg/ml) for 2, 6, and 24 hrs, respectively. The cytoplasmic fractions were extracted and immunoprecipitated (IP) using the anti-phosphotyrosine antibody as described in Materials and Methods. The Tyr-IP complexes were subjected to Western blot using the mouse monoclonal antibody against PSF. Total mouse IgG was detected as an internal loading control. CON: the IP control without primary antibody incubation; 0 h: the control HBMECs without IbeA incubation.

As shown in Figures 4 and 5, PSF might contribute to nuclear translocation of NF-κB. Thus, it is interesting to examine whether IbeA-induced pathogenicities, activation of the key signaling molecules (e.g., ERK and IKK) and nuclear translocation of NF-κB could be blocked by knockdown of PSF. IbeA+ *E. coli* K1 penetration (Figure 7A) and PMN transmigration across HBMECs (Figure 7B) were significantly inhibited by suppression of PSF expression when compared to that of the *ibeA* mutant ZD1. Since PSF is mainly present in the nuclei of resting cells and recruited to the cell surface or raft microdomains upon stimulation with IbeA+ *E. coli* K1, it is necessary to examine its distribution in both the cytoplasmic and nuclear fractions. We performed siRNA-mediated knockdown of PSF in HBMECs to examine the role of this protein in IbeA+ *E. coli*-induced NF-κB signaling. As shown in Figure 7C, cytoplasm and nucleus were completely devoid of PSF in siRNA-treated HBMECs. PSF siRNA was able to completely inhibit Erk1/2 and IKK α/β phosphorylation, IκBα degradation, and NF-κB translocation to the nucleus, suggesting that PSF plays an essential role in the signals for NF-κB activation/translocation that are conveyed between the cytoplasm and nucleus. It has been suggested that the cytoplasmic relocalization of PSF is regulated by tyrosine phosphorylation [43]. We, therefore, examined whether IbeA was able to induce tyrosine phosphorylation of PSF using the Co-IP assay with an anti-phosphotyrosine antibody. As shown in Figure 7D, tyrosine phosphorylation of PSF was significantly increased upon the prolonged IbeA stimulation, which was correlated with the increased PSF relocalization or expression shown in Figure 3B, 4A, 5C and S1B. E44-induced PSF relocalization was significantly reduced in HBMECs transfected with vimentin siRNA (Figure 3B). Recruitment of PSF was also remarkably suppressed in HBMECs transduced with the vimentin head domain deletion construct upon stimulation with IbeA (Figure 5C) and E44 (Figure S1B). These results suggest that regulation of PSF by tyrosine kinases and vimentin may play a role in IbeA-induced NFκB activation and translocation.

### IbeA+ *E. coli* induced proteasomal degradation is inhibited by siRNA-mediated knockdown of vimentin and VH domain deletion

**Figure 8:**
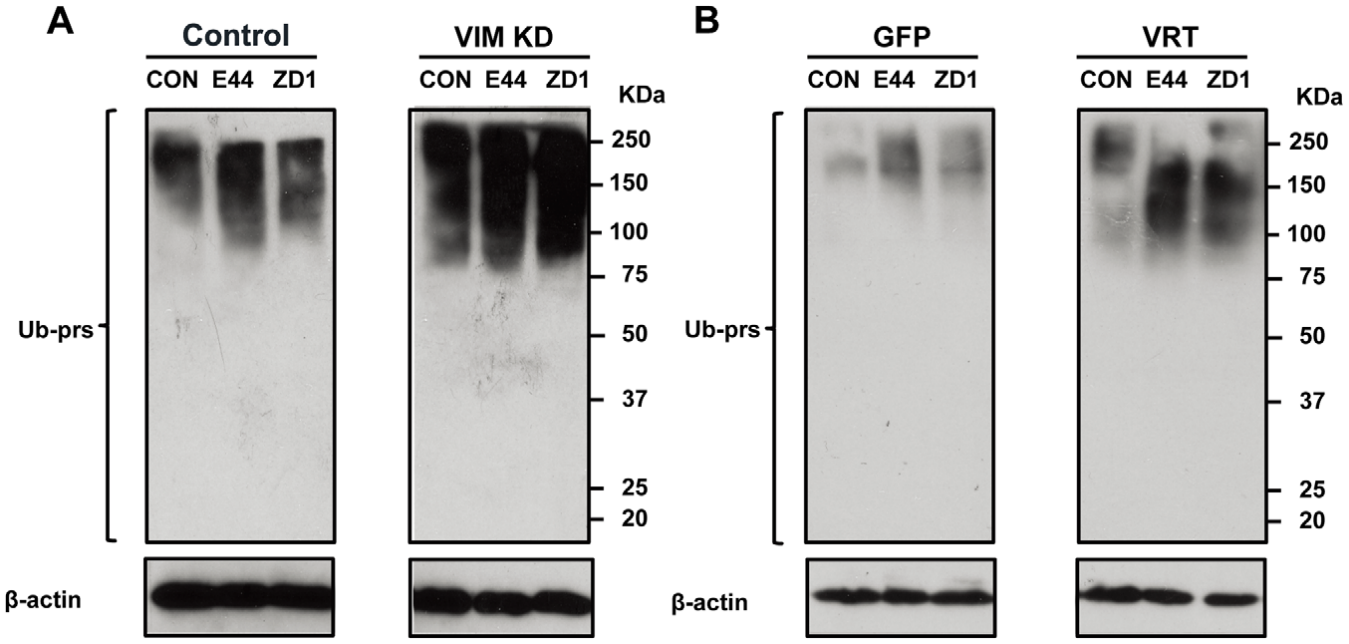
Inhibition of IbeA+ *E. coli* K1-induced proteasomal degradation by knockdown of vimentin and VH domain deletion. Immunoblotting analysis of polyubiquitinylated proteins (Ub-prs): (**A**) HBMECs with siRNA-mediated knockdown of vimentin; and (**B**) HBMECs transduced with the lentivirus constructs expressing GFP–VRT and GFP. In both (A) and (B), all the cells were incubated with or without *E. coli* K1 strains (E44 and ZD1, 10^7^/ml) for 2 h. The Ub-prs were detected in cytoplasmic fractions to determine the proteasomal degradation as described in Materials and Methods. β-actin was used as an internal loading control. In all experiments, untreated HBMECs (UNT) were taken as controls.

Our previous study demonstrated that the proteasome-regulated NF-κB played an important role in IbeA/vimentin-induced expression of adhesion molecules and transmigration of PMNs across HBMECs. WFA (withafferin A), a dual inhibitor of vimentin [4], [44] and the ubiquitin proteasome pathway [45]–[46], could block IbeA-induced upregulation of ICAM-1/CD44 and activation/translocation of NF-κB through a vimentin-dependent mechanism [5]. Vimentin has been implicated in the stabilization of the Scribble polarity protein by protecting it from proteasomal degradation [45]. In order to further examine the role of vimentin in the IbeA-induced proteasomal degradation, we examined the colocalization of vimentin and polyubiquitinylated proteins using immunofluorescence microscopy. As shown in Figure S3, the polyubiquitinylated proteins were clustered and colocalized with vimentin around the perinuclear region upon stimulation with the IbeA protein or IbeA+ *E. coli* (E44), but not in the untreated cells or the cells treated with ZD1. The polyubiquitinylated proteins also aggregated to small particles to colocalize with vimentin on the cell membranes (indicated by arrows), which were called aggresomes and play an important role in the proteasomal degradation [47]. These results suggest that vimentin might directly bind to polyubiquitinylated proteins to exert its protection and inhibit their degradation. The immunoblotting analysis of polyubiquitinylated proteins in HBMECs was consistent with the microscopy examination, which showed that more polyubiquitinylated proteins in HBMECs treated with the control siRNA were induced by IbeA+ *E. coli* K1 than ZD1 or the non-treated control (left panel in Figure 8A). However, siRNA-mediated knockdown of vimentin resulted in stronger polyubiquitinylation than the control siRNA for each treatment, suggesting that vimentin could exert a protective role in proteasomal degradation (Figure 8A). Since the VH domain has been demonstrated as the scaffold region for many signaling proteins, we therefore hypothesized that the VH domain might be required for its protection role in proteasomal degradation. To test this hypothesis, the GFP–VRT transductant with the VH domain deletion was used to examine the polyubiquitinylated proteins in HBMECs treated with E44 or ZD1. As shown in Figure 8B, the GFP–VRT transductant contained considerably more polyubiquitinylated proteins when compared to the GFP transductant. These results indicated that the vimentin head domain is essential for its protective role in proteasomal degradation. PDZ-containing proteins have been implicated in the regulation of NF-κB signaling [48]. Since Scribble, a PDZ protein, has been shown to be stabilized by vimentin, we examined whether Scribble contributed to IbeA+ *E. coli*-induced invasion and PMNs transmigration across HBMECs. Effects of siRNA-mediated knockdown of vimentin and Scribble on IbeA-induced pathogenicities were investigated. As shown in Figure S4A and S4B, the knockdown of both proteins could equally suppress IbeA-mediated bacterial invasion and PMNs transmigration, but not in the cells treated with ZD1. These results implied that Scribble may contribute to IbeA-induced NF-κB signaling.

## Discussion

In this report, we show that NF-κB signaling induced by meningitic *E. coli* K1 requires two IbeA-binding proteins, the primary receptor vimentin and the co-receptor PSF. Our previous studies have demonstrated that the specific interaction between IbeA and its primary receptor vimentin is the upstream signaling event, which is required for the caveolae/lipid raft-dependent entry of *E. coli* K1 into HBMECs [4]. Subsequently, the co-receptor PSF and activated signaling molecules (e.g., ERK, caveolin-1) are recruited to the lipid raft microdomains. Furthermore, ERK, which is able to communicate a signal from a receptor on the cell surface to the DNA in the nucleus, was shown to be important for NF-κB activation in response to Ras activation [36] or anthracycline drug treatment [37]. The current report shows that vimentin forms a complex with IκB, NF-κB and tubulins in the resting cells. A dissociation of this complex and a simultaneous association of PSF with NF-κB could be increased by IbeA in a time-dependent manner. IKK α/β phosphorylation is completely abolished in HBMECs lacking vimentin and PSF. ERK activation is entirely dependent on PSF. These findings suggest that vimentin and PSF cooperatively contribute to IbeA-induced cytoplasmic activation and nuclear translocation of NF-κB. PSF is essential for the translocation of NF-κB and ERK to the nucleus.

The major intermediate filament protein vimentin is an important marker of EMT (epithelial–mesenchymal transition). The EMT phenotypes are usually associated with embryonic development and the malignant conversion of epithelial tumor cells [21], [49]. The EMT phenotype in Ras-transformed epithelial cells is suppressed by inhibition of NF-κB signaling. Blockage of NF-κB is positively correlated with the suppression of vimentin expression and inhibition of the invasive phenotype of prostate cancer cells, suggesting that EMT may be used as a predictor of certain cancers [50]. In concurrence with these findings, IbeA-mediated PMN transmigration can be enhanced by upregulation of vimentin through NF-κB activation [5]. Vimentin expression may be upregulated by activated NF-κB, which binds to a regulatory element (enhancer) in the promoter of the human vimentin-encoding gene [51]. Furthermore, the regulatory role of vimentin in NF-κB activation has been implicated in our recent studies on the effects of vimentin blockage on gene expression, which shows significant inhibition of NF-κB in the nuclei of HBMECs lacking vimentin [5]. Here, we have further demonstrated that vimentin could contribute to regulation of NF-κB by forming a complex with IκB, NF-κB and tubulins in the resting cells. This complex is dissociated upon the prolonged stimulation with IbeA. As reported by Georgatos *et al.* in 1985 [52]–[53], oligomeric species of vimentin bind to human erythrocyte membranes through its head domain-mediated interactions with ankyrin. Since IκB proteins contain multiple ankyrin repeats [54], it is most likely that the head domain of vimentin binds to the ankyrin domains of the IκB proteins. Our results have demonstrated for the first time that the vimentin head domain is essential for its interactions with IκB/NF-κB. It remains to be determined whether this binding requires phosphorylation or other modifications of vimentin. There are two different types of vimentin proteins, the insoluble (e.g., receptors) and the soluble (e.g., secreted), which contribute to bacterial pathogenesis. Vimentin on the surface of BMEC functions as the primary receptor for IbeA and plays an essential role in IbeA+ *E. coli* K1-induced NF-κB signaling, bacterial invasion and PMN migration across the BBB [4]–[5]. However, the soluble vimentin, which can be modified and secreted by macrophages [55] and EC [56], may be involved in microbial infections in a manner similar to the soluble receptors. Their exact relationship with pathogen-induced signaling may warrant further studies. Microtubules may also interact with IκB proteins in the same way as vimentin because they bind to ankyrin domain-containing proteins [57]–[58]. As shown in our previous studies, IbeA-induced NF-κB activation and translocation are abolished by the blockage of vimentin (whithaferin A) and the ubiquitin proteasome system (UPS) (PS-341) [5]. These blockages resulted in accumulation of ubiquitinated proteins in the cytosol, suggesting the involvement of vimentin and UPS. The current studies show that IbeA-induced *E. coli* K1 invasion and PMN transmigration across HBMECs are significantly blocked by siRNA-mediated knockdown of Scribble, a PDZ domain-containing protein regulated by vimentin [45], suggesting that vimentin contributes to IbeA-induced activation and nuclear translocation of NF-κB in coordination with UPS and Scribble. The PDZ protein, DLIM2, has been recently identified as a novel regulator of NF-κB [48]. Defining the role of vimentin, UPS and Scribble in IbeA-induced signaling may add a new layer to the mechanics of NF-κB regulation.

PSF exhibits multiple functions, including the binding of nucleic acids (DNA and RNA) and proteins, DNA pairing, promotion of pre-mRNA splicing, and transcriptional regulation [35]. PSF has been shown to be one of the key factors mediating the posttranscriptional regulation of HIV-1 and causes a dose-dependent inhibition of virus production in cell culture [59]. It has been clearly established that PSF also contributes to the regulation of gene expression mediated by several nuclear hormones (e.g., thyroid hormone, progesterone, and androgens) receptors via different mechanisms, including inhibition of DNA binding, proteosomal receptor degradation, and/or recruitment of coactivators such as the histone deacetylase (HDAC) complex [23]. Transcriptional activity of several steroid receptors could be blocked by overexpression of PSF through direct protein-protein interactions with several transcriptional regulators [60]. Association of PSF with pERK was recently found to be highly regulated [23]. Insulin-like growth factor-I could dissociate the pERK-PSF complexes, which would then result in integration of many signaling events to allow dissociated pERK to access, phosphorylate, and activate Sp1, thereby driving steroidogenesis [23]. We have demonstrated that siRNA-mediated knockdown of PSF could completely abolish phosphorylation of ERK and IKK, and nuclear translocation of NF-κB, suggesting that PSF association with pERK and pIKK may be one of the mechanisms contributing to the regulation of IbeA-induced NF-κB signaling. Recently, we demonstrated that p54nrb, a PSF protein partner, could contribute to IbeA+ *E. coli*-induced pathogenicities [61]. Due to their dual RNA/DNA-binding properties and the ability to interact with several protein counterparts, PSF and p54nrb might contribute to multiple gene regulation processes, which may play a crucial role in *E. coli* K1-induced activation and nuclear translocation of NF-κB. Thus, it can be speculated that IbeA/vimentin/PSF interactions may represent an important mechanism responsible for a coordinated modulation of both bacterial and host factors that contribute to bacterial invasion and PMN transmigration. Further work is needed to examine how vimentin and PSF cooperatively contribute to IbeA-induced NF-κB signal transduction.

In this study we also show that functional microtubules are required for the nuclear transport of transcription factor NF-κB in HBMECs upon stimulation with IbeA. Several microtubule inhibitors (colchicine, vincristine, nocodazole) that can inhibit tubulin polymerization decreased IbeA-induced nuclear translocation of active NF-κB into the nucleus. Two of these inhibitors, colchicines and vincristine, could also block phosphorylation of ERK and IKK. It has been shown that two major cytoskeletal systems, actin filament and microtubule, are able to regulate the NF-κB system [48], [62]–[63]. It has been well established that there are interactions or crosstalks between intermediate filaments and microtubules [64]. Their close association in several cell types is demonstrated by electron-microscopy studies. For example, these two kinds of cytoskeletons form closely associated parallel arrays throughout the cytoplasm of fibroblasts. A specific subset of stable, detyrosinated microtubules (Glu-MTs) are shown to be involved in these associations [64]. A crosstalk between intermediate filaments and microtubules is suggested in the current studies as β-tubulin binds to the head domain of vimentin. Understanding the molecular basis of the crosstalk between vimentin intermediate filaments and microtubules may be essential for determining the mechanisms that underlie NF-κB activation.

In summary, the current studies with IbeA+ *E. coli* K1 infection in HBMECs show that vimentin and PSF play an important role in the pathogenesis of meningitic infection by contributing to modulation of the early and late events of NF-κB signaling in a coordinated manner. Cooperative contributions of vimentin and PSF to IbeA-induced cytoplasmic activation and nuclear translocation of NF-κB may represent a new paradigm in pathogen-induced signal transduction and lead to the development of novel strategies for prevention and treatment of bacterial meningitis.

## Materials and Methods

### Ethics statement

All research involving human participants has been approved by the Institutional Review Board (IRB) of Children's Hospital Los Angeles (CHLA). Human polymorphonuclear leukocytes (PMNs) were isolated from heparin anticoagulated, human peripheral venous blood of healthy adult volunteers using the standard Ficoll-Hypaque method in accordance with the protocol approved by the CHLA Committee on Clinical Investigations (CCI), which is the IRB for Human Subjects at Saban Research Institute of CHLA. This protocol has been granted a waiver of informed or signed consent per 45 CFR 46.116(d) and a waiver of HIPAA authorization per the Privacy Rule (45 CFR Part 160 and Subparts A and E of Part 164). No minors/children participants were involved in our studies. The animal study was performed in strict accordance with the recommendations in the Guide for the Care and Use of Laboratory Animals of the National Institutes of Health. Our protocols were approved by the Institutional Animal Care and Use Committee (IACUC) of The Saban Research Institute of CHLA (Permit number: A3276-01). All surgery was performed under anesthesia with ketamine and lidocaine, and all efforts were made to minimize suffering.

### Chemicals and reagents

Dextran, polymyxin B resin, colchicine, vincristine, nocodazole were purchased from Sigma-Aldrich (St. Louis, MO, USA). Restriction endonucleases and T4 DNA ligase were obtained from New England Biolabs (Beverly, MA, USA). CAPE (caffeic acid phenyl ester) and PD098059 were purchased from Calbiochem (Darmstadt, Germany). Two peptides, ERK89 (vimentin binding domain: RAPTIEQMKDVYIVQ) and ERK312 (control peptide: EQYYDPSDEPIAEAP), were synthesized by GenScript (Piscataway, NJ, USA). Plasmid DNA was extracted by using the MiniPrep kit (Qiagen Inc., Chatsworth, CA, USA). Mouse monoclonal antibodies against GFP (T007) and His (T001) were gifts from Epitope Biotech Inc. (Burnaby, Canada). All other primary antibodies (Ab) and related reagents were purchased from the following commercial sources: (A) rabbit polyclonal antibodies against β-actin (sc-130657), vimentin (H84) (sc-5565), NF-κB (p65) (sc-109), and GFP (sc-8334), and mouse monoclonal antibodies against vimentin (V9) (sc-6260), and β-tubulin (sc-5274), and Protein G and Protein A–agarose beads from Santa Cruz Biotechnology (Santa Cruz, CA); (B) a mouse monoclonal antibody against PSF (P2860) from Sigma-Aldrich(St. Louis, MO, USA); (C) rabbit polyclonal antibodies against phospho-ERK1/2 (Thr202/Tyr204) (#4370), phospho-IKK α/β (Ser176/180) (#2697), β-tubulin (#2146), IKKβ (#2678), and a mouse monoclonal antibody against IκBα (#4814) from Cell Signaling Technology (Danvers, MA); (D) a polyubiquitinylated proteins mAb (FK1, BML-PW8805-0500) from Endo Life Sciences (Farmingdale, NY); (E) a rat anti-mouse Ly-6G (Gr-1) Ab, from eBiosciences (San Diego, CA); and (F) a rabbit polyclonal α7 nAChR Ab from Genescript (Piscataway, NJ). Transwell filters (3 mm pore size, 6.5 mm diameter), blood plates and a mouse monoclonal antibody against phosphotyrosine proteins were purchased from BD Biosciences (San Jose, CA). Other chemicals were purchased from Sigma-Aldrich unless otherwise noted.

### Protein expression and purification

pET17A for expression of His6–IbeA was transformed into *E. coli* BL21(DE3) cells. Expression and purification of recombinant proteins in *E. coli* BL21 (DE3) cells were carried out according to the manufacturer's instructions (EMD Bioscience). Briefly, purification of His6–IbeA was performed under the denaturing condition (8 M urea) using Ni-NTA (Ni^2+^- nitrilotriacetate)–agarose columns. The eluted protein was dialyzed against decreasing concentrations of urea in the same buffer as described previously [19]. The purified IbeA protein was incubated with polymyxin B resin to remove the potentially contaminated lipopolysaccharide (LPS) [5]. The pull-down assay was carried out as described previously [4]. Briefly, the Ni-NTA resin with or without (control) purified native His–vimentin was incubated with total cell lysates from HBMEC on a rocking platform overnight at 4°C, and then washed with wash buffer for five times. The pull-down complex was released from Ni-NTA resin by boiling in 2×SDS sample buffer, and then immunoblotted with mouse antibodies against the His tag, vimentin (V9) and IκBα, respectively.

### Brain endothelial cells, *E. coli* strains and Invasion

HBMECs were kindly provided by Dr. Kwang S. Kim (John Hopkins University) [65] and cultured as described previously [2]–[8], [66]. HBMECs were routinely cultured in RPMI 1640 medium (Mediatech, Herndon, VA) containing 10% heat inactivated fetal bovine serum, 10% Nu-serum, 2 mM glutamine, 1 mM sodium pyruvate, essential amino acids, vitamins, penicillin G (50 mg/ml) and streptomycin (100 mg/ml) at 37°C in 5% CO2. All the phenotypes of HBMECs were described as previously [5]. E44 is a rifampin-resistant derivative of *E. coli* K1 strain RS218 (serotype 018:K1: H7) [19], [65]. ZD1 is an *ibeA* in-frame-deletion mutant of E44 [19]. GFP (green fluorescent protein)-expressing E44 was made by transformation with pHC60 encoding the *gfp* gene that is constitutively expressed [67]. All strains were grown at 37°C in brain–heart infusion (BHI) media supplemented with rifampin (100 µg/ml) and/or tetracycline (10 µg/ml) if required. *E. coli* DH5a was used as the host strain for subcloning. *E. coli* invasion was performed as described previously [19]–[20]. The inhibitor CAPE was pre-incubated with the HBMEC monolayers for 1 h at 37°C before addition of bacteria and present throughout the invasion experiment until the medium was replaced with experimental medium containing gentamicin (100 µg/ml) for 1 h of incubation. The released intracellular bacteria were enumerated by plating on sheep blood agar plates. Cell viability was routinely verified by the trypan blue staining assay. Results were expressed as relative invasion percentage of invasion in comparison to that of untreated HBMECs.

### PMN isolation and transmigration assay

Neutrophils were isolated by dextran sedimentation of leukocytes followed by Ficoll– Hypaque density gradient centrifugation of PMN and hypotonic lysis of erythrocytes as previously described [68]–[69]. PMN suspensions in experimental medium (EM; containing 49% M199, 49% Ham's F12, 1 mM sodium pyruvate and 2 mM L-glutamine) were used for transmigration assays as described previously [5], [68]–[69]. Briefly, the confluence of the HBMEC monolayers in transwell filters (3.0-mm pore size, 6.5-mm diameter, BD Biosciences) was confirmed by light microscopy before the start of the assay. The transwell filters were transferred to clean 24-well plates, and washed twice with EM. The HBMEC monolayers were pre-incubated with different doses of CAPE (both in the upper and lower chambers) for 60 min, and stimulated with *E. coli* K1 strains (10^6^) in the lower chambers with 0.8 ml EM for 2 h. Then, PMN (1×10^6^ cells in 0.2 ml of EM) were added to the upper chamber and allowed to migrate over for 4 h. The CAPE was present throughout the PMN transmigration experiment until the end. At the end of the incubation, migrated PMN cells were collected from the lower chamber and counted in a blinded-fashion using a hemacytometer. Final results of PMN transmigration were expressed as the percentage of PMN across the HBMEC monolayers.

### Mouse model of *E. coli* meningitis

The neonatal mice were randomly divided into two groups (5 mice each group). CAPE in 50% DMSO was administered for 3 days by intraperitoneal (i.p.) injection (10 mg/kg body weight daily) [70]. The control group also received equal volume of 50% DMSO at the same time. At 10 days of age, all pups received *E. coli* K1 strain E44 (10^5^ CFU) by i.p. injection. Eighteen hours after *E. coli* inoculation, animals were anaesthetized with ketamine and lidocaine. Sectioning of brain tissues and sampling of blood and CSF were carried out as described previously [5]. To determine PMN transmigration across the BBB, CSF samples were stained with a FITC-conjugated rat anti-mouse Ly-6G (Gr-1) antibody and counted under the fluorescence microscope. Albumin concentrations in the CSF samples were determined using a mouse Albumin ELISA kit from Bethyl laboratories (Montgomery, TX) according to the manufacturer's instructions. To measure soluble NF-κB, five µl of the CSF samples were taken and subjected to ELISA using rabbit polyclonal antibodies against NF-κB (p65) from Santa Cruz Biotechnology according to the manufacturer's instructions.

### Overexpression of vimentin head (VH) (RT deletion mutant) and RT (VRT) (VH deletion mutant) domains in HBMECs transduced with lentivirus constructs

The lentivirus constructs expressing GFP-VRT or GFP were generated as described previously [4]. To make a lentivirus construct expressing GFP-VH, VH cDNA was amplified with two primer pairs: VH5 (5-TCCGGATCCATGTCCACCAGGTCCGTG-3) and VH3 (5-ATGAATTCCTCGGTGTTGATGGCGTC-3) and then cloned into pEGFP-C1 (BD Biosciences) at the BglII and EcoRI sites in the C-terminus of GFP. The fragment GFP–VH was double-cut with the NheI/SalI sites and cloned into pRRLsinhCMV (lentivirus vector) at the XbaI/SalI sites. The lentivirus packaging, production and transduction in HBMECs were described by Chen et al. [71]. After 48 h, conditioned medium was collected and filtered, and the lentivirus stock was stored at−70°C. The 60–80% confluent HBMECs were infected with the lentivirus. The GFP expression was examined under a fluorescence microscope at 48 h incubation after lentivirus transduction. The transduced HBMECs with 70–90% expression of GFP were subjected to western blotting, fluorescence microscopy examination, and co-immunoprecipitation as described below.

### Vimentin, PSF and Scribble knockdown experiments

Knockdown experiments were performed using the human vimentin, PSF **and Scribble** siRNA (small interfering RNA) kits from Santa Cruz Biotechnology according to the manufacturer's instructions. siRNA duplex (60 pmol) was diluted with the transfection medium and incubated with HBMECs. The cells were incubated for 18–24 h. After replacing with fresh medium and incubating for an additional 24 h, cells were used for invasion assays, PMN transmigration assays, and western blotting analysis.

### Immunoblotting analysis

To assess IbeA protein or IbeA+ *E.coli* K1 induced NF-κB activation in HBMECs, endothelial cell monolayers were grown on 60-mm plates. Confluent HBMEC monolayers were incubated with E44, ZD1 (10^7^/ml), or IbeA (0.1 µg/ml) for 30 min or 2 hours. For the blocking assays with inhibitors, HBMEC monolayers were pre-incubated with or without CAPE, PD098059, colchicine, vincristine, nocodazole and the ERK peptides for 60 min, and then simulated with E44, ZD1 or IbeA as described above. HBMECs with siRNA transfection or lentivirus transduction were also stimulated with E44, ZD1 or IbeA as described above. After the completion of incubation, cytoplasmic and nuclear proteins of HBMECs were extracted with lysis buffer supplied with 100 nM okadaic acid and 1 mM Na_3_VO_4_ as described previously [46]. Both cytoplasmic and nuclear proteins were mixed with SDS buffer, heated and subjected to sodium dodecyl sulfate polyacrylamide gel electrophoresis (SDS–PAGE). Separated proteins were transferred to nitrocellulose membrane by semi-dry blotting. After overnight blocking with 5% milk in PBS with 0.1% Tween 20, membranes with cytoplasm proteins were probed with antibodies against phospho-ERK1/2 (Thr202/Tyr204) (0.2 µg/ml), phospho-IKK α/β (0.4 µg/ml), IκBα (0.2 µg/ml, Cell Signaling Technology), vimentin (V9, 0.2 µg/ml), α7 nAChR (0.1 µg/ml), PSF (0.2 µg/ml), β-actin (0.1 µg/ml) for 2 hours, respectively. The membranes with nuclear proteins were probed with antibodies against NF-κB (p65) (0.4 µg/ml), PSF (0.2 µg/ml), and β-actin (0.1 µg/ml) for 2 h, respectively. For proteasome activity examination, the membranes with cytoplasmic proteins were incubated with a mAb against polyubiquitinylated proteins (FK1, 0.2 µg/ml) for 2 h. The washed membranes were incubated with a horseradish peroxidase (HRP)-conjugated secondary antibody for 1 h and then visualized using an enhanced chemiluminescence procedure (Roche Applied Science, Indianapolis, IN).

### Co-immunoprecipitation

HBMECs grown in 100-mm-diameter dishes were washed with cold PBS for 2 times after stimulated with IbeA (0.1 µg/ml) for different times (2, 6, and 24 h). The cells were harvested, and the cytoplasmic and nuclear proteins were extracted with 500 µl of lysis buffer as described above. One hundred µl of lysates were taken from each fraction, and mixed with SDS sample buffer to serve as positive controls. For Co-immunoprecipitation, the cytoplasmic fractions (200 µl) were mixed with primary antibodies against vimentin (V9) (4 µg/ml), NF-κB (p65) (4 µg/ml), and phosphotyrosine proteins (4 µg/ml), respectively. For lentivirus transduced HBMECs, the cytoplasmic proteins were also extracted from untreated cells as described above, and mixed with the mouse monoclonal antibody against GFP (4 µg/ml). All the mixtures were incubated with gentle rocking overnight at 4°C, and then added with Protein G–or Protein A–agarose beads for additional gentle rocking for 1–3 h at 4°C. The lysates without incubation with primary antibodies were also incubated with Protein G–or Protein A–agarose beads to serve as negative controls. After centrifugation five times at 8000 g for 30 s and washing with lysis buffer, the pellet was resuspended with 30 µl of 3×SDS sample buffer. All the samples were analyzed by SDS/PAGE (12.5%) and immunoblotted as described above with the antibodies against IKKβ (0.4 µg/ml), β-tubulin (0.4 µg/ml; the rabbit Ab for the vimentin Co-IP complex and the mouse Ab for the p65 Co-IP complex), IκBα (0.2 µg/ml), NF-κB (p65) (0.4 µg/ml), α7 nAChR (0.1 µg/ml), vimentin (0.2 µg/ml; V9 for the p65 Co-IP complex and H84 for the vimentin Co-IP complex), PSF (0.2 µg/ml), GFP (0.2 µg/ml; the rabbit Ab), and β-actin (0.1 µg/ml), respectively.

### Immunofluorescence microscopy

HBMECs transduced with or without lentivirus constructs were grown in eight-well chamber slides coated with collagen and incubated for 2 days, and stimulated with IbeA (0.1 µg/ml) or *E. coli* K1 strains with or without GFP expression. The HBMEC monolayers were then washed with PBS and fixed with 4% (w/v) paraformaldehyde for 30 min at room temperature (25°C). After blocking with 5% (w/v) BSA in PBS, the cells were stained with the monoclonal antibody against vimentin (V9) (2 µg/ml)/FITC, a polyclonal antibody against NF-κB (p65) (2 µg/ml,)/rhodamine, a polyclonal antibody against β-tubulin (2 µg/ml)/rhodamine, or a monoclonal antibody (IgM) against polyubiquitinylated proteins (FK1, 2 µg/ml)/rhodamine. The cells were immersed with mount medium containing 4′-6-diamidino-2-phenylindole (DAPI) solution, and examined under a fluorescence microscope at the Congressman Dixon Cellular Imaging Core Facility, Children's Hospital Los Angeles. To ensure the fluorescence strength of each treatment in a comparable manner, all the images were acquired with the same parameters.

### Statistical analysis

All results given are means+−S.D. of triplicate determinations in invasion and PMN transmigration assays. Raw data were entered into Microsoft® Excel files and were analyzed using the statistical package. Software GraphPad Prsim 5.0 was used for analysis of data from the animal experiments. P ANOVA and co-variates were followed by a multiple comparison test. <0.05 was considered to be significant.

### Database

The protein access codes in Swissprot database are listed as follows: vimentin, Homo sapiens (Human), P08670; Transcription factor p65 [NF-κB (p65)], Homo sapiens (Human), Q04206; NF-kappa-B inhibitor alpha [IκBα], Homo sapiens (Human), P25963; Extracellular signal-regulated kinase 1 [ERK1], Homo sapiens (Human), P27361; Extracellular signal-regulated kinase 2 [ERK2], Homo sapiens (Human), P28482; Tubulin beta chain [β-tubulin], Homo sapiens (Human), P07437; Inhibitor of nuclear factor kappa-B kinase subunit alpha [IKK α], Homo sapiens (Human), O15111; Inhibitor of nuclear factor kappa-B kinase subunit beta [IKK β], Homo sapiens (Human), O14920; PTB-associated-splicing factor [PSF], Homo sapiens (Human), P23246 ; Neuronal acetylcholine receptor subunit alpha-7 [α7 nAChR or CHRNA7], Homo sapiens (Human), P36544; Protein scribble homolog [Scribble], Homo sapiens (Human), Q14160; β-actin, Homo sapiens (Human), P60709; Green fluorescent protein, [GFP], Aequorea victoria (Jellyfish), P42212.

## Supporting Information

Figure S1 **Vimentin head domain is required for IbeA+** ***E. coli*** **K1 induced NF-κB activation.** (**A**) The cytoplasmic proteins of GFP–VRT and GFP transductant were subjected to Western blot using the rabbit anti-GFP antibody, the V9 antibody against vimentin, and the H84 antibody against vimentin. Band a, GFP–VRT (72 kDa); band b, GFP-VH (37 kDa); band c, GFP (27 kDa); band d, vimentin (55 kDa). β-actin was used as the internal loading control. (**B**) Western blot of GFP–VRT and GFP transductant treated with *E. coli* K1 strains. p-Erk1/2, p-IKK α/β, IκBα degradation and PSF re-localization were examined in cytoplasmic fractions after 30 min of treated with E44 and ZD1. NF-κB (p65) translocation to the nucleus was examined in nuclear fractions after 2 h of incubation with E44 and ZD1. β-actin in both fractions was detected as internal loading controls. CON: control without bacterial stimulation. (**C**) Immunofluorescence images of the GFP–VRT and GFP transductant stimulated GFP-tagged E44 or ZD1 (25 MOI) for 2 h. The cells were double-stained with the rabbit antibody against NF-κB (p65) conjugated to rhodamine (red), and DAPI (blue). Scale bar, 25 µm. Arrows indicated cells with NF-κB (P65) translocation to the nucleus, which was increased in the GFP transductants and reduced in the GFP-VRT-transduced HBMECs.
(TIF)

Figure S2 ***In vitro*** **Pull-down assays to detect binding between vimentin and IκBα.** His–vimentin pull-down assays were performed as described in Methods and Materials. The pull-down complexes were detected by: Coomassie Brilliant Blue (CBB) R-350 staining (**A**) and antibodies against the His6 tag (**B**), vimentin (**C**) and IκBα (**D**).
(TIF)

Figure S3 **Colocalization of IbeA− or IbeA+** ***E. coli*** **K1-induced vimentin clusters with polyubiquitinylated proteins.** Immunofluorescence microscopy was used to examine colocalization of vimentin clusters and polyubiquitinylated proteins after 2 h of stimulation with IbeA (0.1 µg/ml), E44 or ZD1 (25MOI). HBMECs were triple-stained with the V9 antibody against vimentin conjugated to FITC (green), the mouse antibody (IgM) against polyubiquitinylated proteins conjugated to rhodamine (red), and DAPI (blue). The merged images are shown in the right-hand panels (Merge). Arrows indicated polyubiquitinylated proteins in aggregates of small particles, which are colocalized with vimentin on the cell membranes. Scale bar, 50 µm.
(TIF)

Figure S4 **Inhibition of IbeA+** ***E. coli*** **K1-induced invasion and PMN transmigration across HBMECs by knockdown of vimentin and Scribble.** IbeA+ *E. coli* K1-induced invasion (**A**) and PMN transmigration (**B**) were inhibited by siRNA-mediated knockdown of vimentin (VIM) and Scribble (Scrib). The siRNA-mediated knockdown of VIM and Scrib was performed with HBMECs grown in 24-well plates or transwell filters. After 24 h incubation, invasion and PMN transmigration assays were carried out as described in the Materials and Methods. The HBMECs in transwell filters were stimulated with *E. coli* K1 strains (10^6^ CFU) in the lower chambers for 2 h before adding PMN (10^6^) in the upper chambers. Both invasion and PMN transmigration assays were performed in triplicates. Results for invasion are expressed as a relative percentage compared to the penetration rate of E44 in the siRNA control (CON) (set as 100%). Results for PMN transmigration are expressed as the percentage of PMN transmigration of total added PMNs. The control siRNA-transfected HBMECs infected with E44 and ZD1 were taken as the controls (panels A and B). The significant differences regarding to the control were marked by asterisks (*P<0.05; **P<0.01).
(TIF)
