# Supplementary figures and images for: Vimentin and PSF Act in Concert to Regulate IbeA+ E. coli K1 Induced Activation and Nuclear Translocation of NF-κB in Human Brain Endothelial Cells
Source: PLoS One. 2012 Apr 20;7(4):e35862. doi: 10.1371/journal.pone.0035862 (PMC3334993; doi:10.1371/journal.pone.0035862)

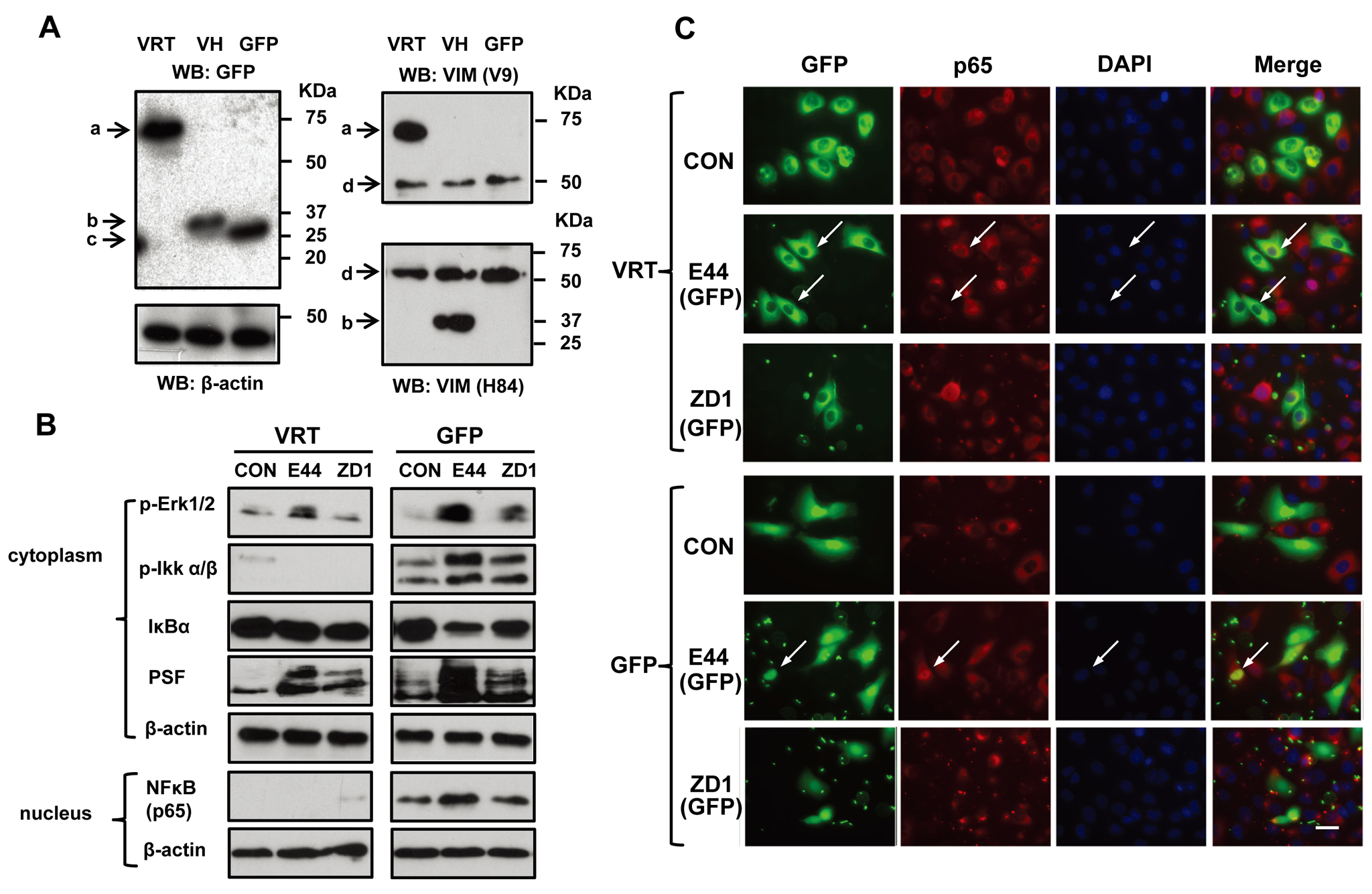

Supplement: Figure S1 — Vimentin head domain is required for IbeA+ E. coli K1 induced NF-κB activation. (A) The cytoplasmic proteins of GFP–VRT and GFP transductant were subjected to Western blot using the rabbit anti-GFP antibody, the V9 antibody against vimentin, and the H84 antibody against vimentin. Band a, GFP–VRT (72 kDa); band b, GFP-VH (37 kDa); band c, GFP (27 kDa); band d, vimentin (55 kDa). β-actin was used as the internal loading control. (B) Western blot of GFP–VRT and GFP transductant treated with E. coli K1 strains. p-Erk1/2, p-IKK α/β, IκBα degradation and PSF re-localization were examined in cytoplasmic fractions after 30 min of treated with E44 and ZD1. NF-κB (p65) translocation to the nucleus was examined in nuclear fractions after 2 h of incubation with E44 and ZD1. β-actin in both fractions was detected as internal loading controls. CON: control without bacterial stimulation. (C) Immunofluorescence images of the GFP–VRT and GFP transductant stimulated GFP-tagged E44 or ZD1 (25 MOI) for 2 h. The cells were double-stained with the rabbit antibody against NF-κB (p65) conjugated to rhodamine (red), and DAPI (blue). Scale bar, 25 µm. Arrows indicated cells with NF-κB (P65) translocation to the nucleus, which was increased in the GFP transductants and reduced in the GFP-VRT-transduced HBMECs. (TIF) [file pone.0035862.s001.tif]

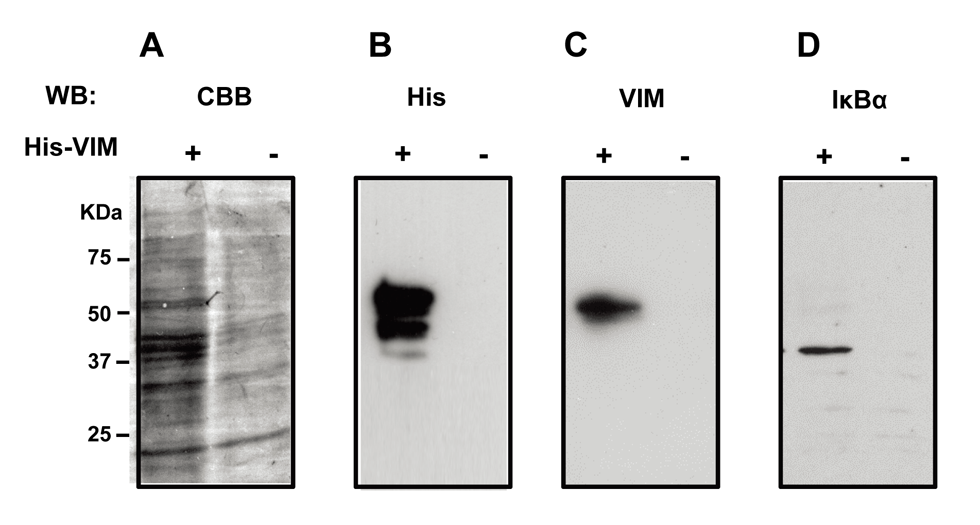

Supplement: Figure S2 — In vitro Pull-down assays to detect binding between vimentin and IκBα. His–vimentin pull-down assays were performed as described in Methods and Materials. The pull-down complexes were detected by: Coomassie Brilliant Blue (CBB) R-350 staining (A) and antibodies against the His6 tag (B), vimentin (C) and IκBα (D). (TIF) [file pone.0035862.s002.tif]

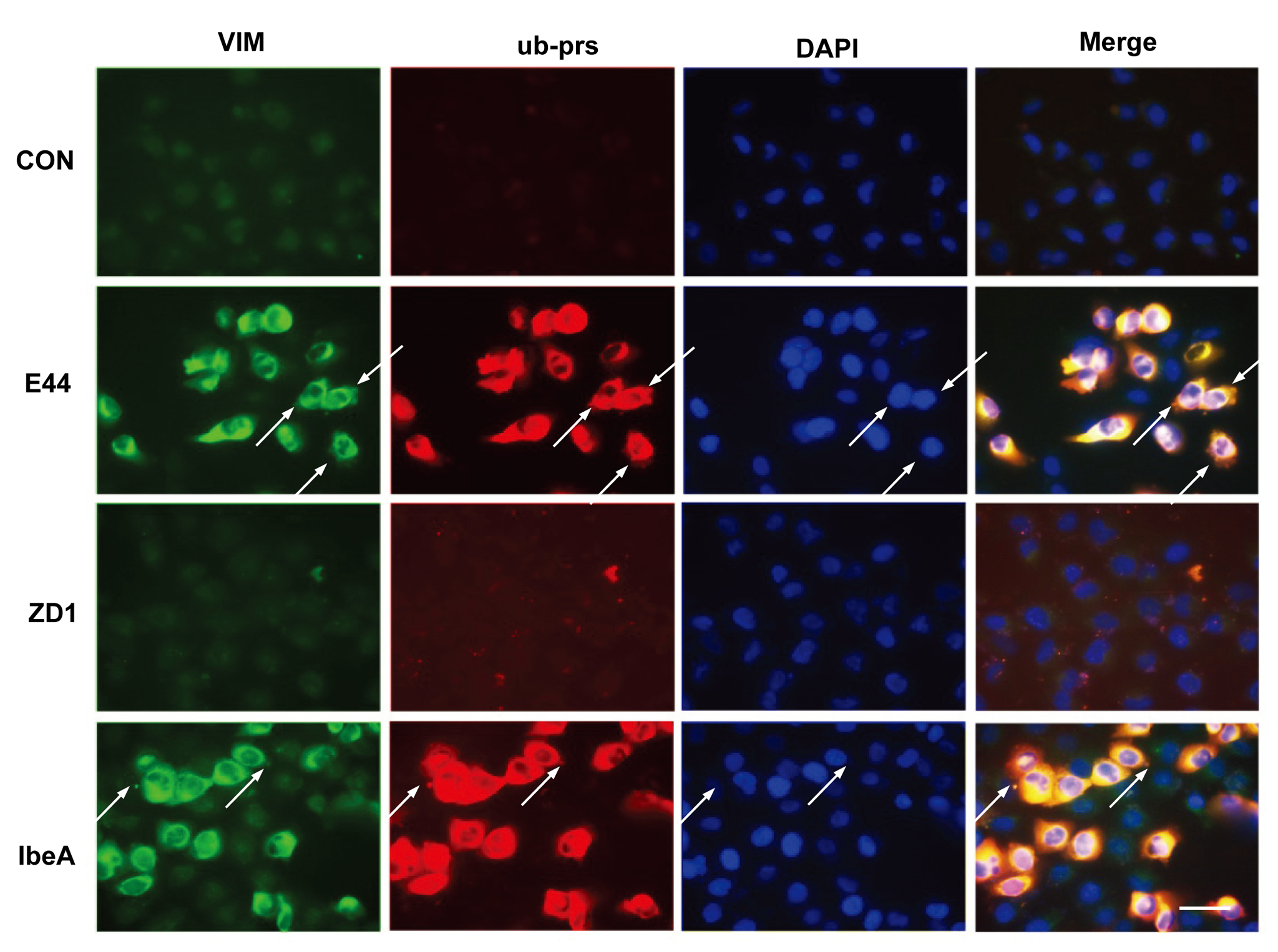

Supplement: Figure S3 — Colocalization of IbeA− or IbeA+ E. coli K1-induced vimentin clusters with polyubiquitinylated proteins. Immunofluorescence microscopy was used to examine colocalization of vimentin clusters and polyubiquitinylated proteins after 2 h of stimulation with IbeA (0.1 µg/ml), E44 or ZD1 (25MOI). HBMECs were triple-stained with the V9 antibody against vimentin conjugated to FITC (green), the mouse antibody (IgM) against polyubiquitinylated proteins conjugated to rhodamine (red), and DAPI (blue). The merged images are shown in the right-hand panels (Merge). Arrows indicated polyubiquitinylated proteins in aggregates of small particles, which are colocalized with vimentin on the cell membranes. Scale bar, 50 µm. (TIF) [file pone.0035862.s003.tif]

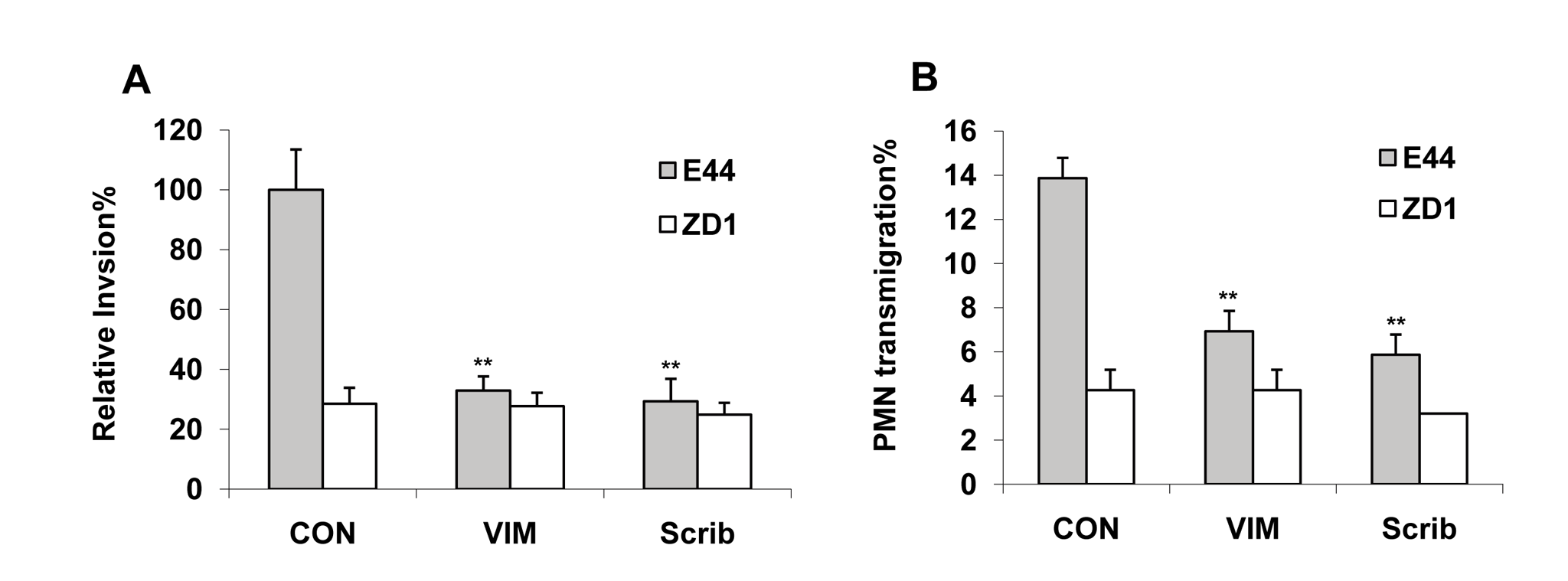

Supplement: Figure S4 — Inhibition of IbeA+ E. coli K1-induced invasion and PMN transmigration across HBMECs by knockdown of vimentin and Scribble. IbeA+ E. coli K1-induced invasion (A) and PMN transmigration (B) were inhibited by siRNA-mediated knockdown of vimentin (VIM) and Scribble (Scrib). The siRNA-mediated knockdown of VIM and Scrib was performed with HBMECs grown in 24-well plates or transwell filters. After 24 h incubation, invasion and PMN transmigration assays were carried out as described in the Materials and Methods. The HBMECs in transwell filters were stimulated with E. coli K1 strains (106 CFU) in the lower chambers for 2 h before adding PMN (106) in the upper chambers. Both invasion and PMN transmigration assays were performed in triplicates. Results for invasion are expressed as a relative percentage compared to the penetration rate of E44 in the siRNA control (CON) (set as 100%). Results for PMN transmigration are expressed as the percentage of PMN transmigration of total added PMNs. The control siRNA-transfected HBMECs infected with E44 and ZD1 were taken as the controls (panels A and B). The significant differences regarding to the control were marked by asterisks (*P<0.05; **P<0.01). (TIF) [file pone.0035862.s004.tif]
